# Exploratory Analysis of Circulating miRNA Signatures in Atrial Fibrillation Patients Determining Potential Biomarkers to Support Decision-Making in Anticoagulation and Catheter Ablation

**DOI:** 10.3390/ijms21072444

**Published:** 2020-04-01

**Authors:** Naoki Kiyosawa, Kenji Watanabe, Yoshiyuki Morishima, Takeshi Yamashita, Naoharu Yagi, Takuto Arita, Takayuki Otsuka, Shinya Suzuki

**Affiliations:** 1 Specialty Medicine Research Laboratories I, Daiichi Sankyo Co., Ltd., Tokyo 140-0005, Japan; 2 Biomarker & Translational Research Department, Daiichi Sankyo Co., Ltd., Tokyo 140-0005, Japan; watanabe.kenji.rv@daiichisankyo.co.jp; 3 Medical Science Department, Daiichi Sankyo Co., Ltd., Tokyo 103-8426, Japan; morishima.yoshiyuki.t4@daiichisankyo.co.jp; 4 Department of Cardiovascular Medicine, The Cardiovascular Institute, Tokyo 106-0031, Japan; yamt-tky@umin.ac.jp (T.Y.); arita@cvi.or.jp (T.A.); otsuka@cvi.or.jp (T.O.); sinsuz-tky@umin.net (S.S.)

**Keywords:** atrial fibrillation, circulating miRNA, biomarker, stroke risk, fibrosis, catheter ablation

## Abstract

Novel biomarkers are desired to improve risk management for patients with atrial fibrillation (AF). We measured 179 plasma miRNAs in 83 AF patients using multiplex qRT-PCR. Plasma levels of eight (i.e., hsa-miR-22-3p, hsa-miR-128-3p, hsa-miR-130a-3p, hsa-miR-140-5p, hsa-miR-143-3p, hsa-miR-148b-3p, hsa-miR-497-5p, hsa-miR-652-3p) and three (i.e., hsa-miR-144-5p, hsa-miR-192-5p, hsa-miR-194-5p) miRNAs showed positive and negative correlations with CHA_2_DS_2_-VASc scores, respectively, which also showed negative and positive correlations with catheter ablation (CA) procedure, respectively, within the follow-up observation period up to 6-month after enrollment. These 11 miRNAs were functionally associated with TGF-β signaling and androgen signaling based on pathway enrichment analysis. Seven of possible target genes of these miRNAs, namely *TGFBR1*, *PDGFRA*, *ZEB1*, *IGFR1*, *BCL2*, *MAPK1* and *DICER1* were found to be modulated by more than four miRNAs of the eleven. Of them, TGFBR1, PDGFRA, ZEB1 and BCL2 are reported to exert pro-fibrotic functions, suggesting that dysregulations of these eleven miRNAs may reflect pro-fibrotic condition in the high-risk patients. Although highly speculative, these miRNAs may potentially serve as potential biomarkers, providing mechanistic and quantitative information for pathophysiology in daily clinical practice with AF such as possible pro-fibrotic state in left atrium, which would enhance the risk of stroke and reduce the preference for performing CA.

## 1. Introduction

Atrial fibrillation (AF) is the most common cardiac arrhythmia. Pathophysiological mechanisms of AF are associated with electrical and structural remodeling, including atrial dilatation, cellular hypertrophy, dedifferentiation, fibrosis, cell death and inflammation [1,2,3]. AF is associated with increased risk of stroke [4], and direct oral anticoagulants (DOAC) has been preferentially used over warfarin to prevent stroke [5].

CHA_2_DS_2_-VASc score is useful for stratifying AF patient with risk of stroke [6]. There is sufficient evidence that male patients with a CHA_2_DS_2_-VASc score ≥2 and female patients with a score ≥3 should be anticoagulated [7,8]. However, CHA_2_DS_2_-VASc score is a point-based score taking account of selected risk factors (i.e., congestive heart failure, hypertension, age 65–74 years, diabetes, female sex and vascular disease, which all count for 1 point, and previous transient ischemic stroke/stroke or age ≥75 years, which count for two points), and there are differences in their impacts for the stroke risk. For instance, the age 65–74 years was reported to be consistently associated with the highest risk among the risk factors in AF patients with CHA_2_DS_2_-VASc score of 1 [7]. In addition, other than the risk factors adopted in CHA_2_DS_2_-VASc score, chronic kidney disease with a creatinine clearance <60 mL/min was shown to increase risks for stroke or systemic embolism more than 8-fold in patients with a CHA_2_DS_2_-VASc score of 1 [9]. Thus, novel biomarkers are desired to provide mechanistic and quantitative information for patients’ pathophysiological conditions, which would improve patient management on top of CHA_2_DS_2_-VASc score. 

Catheter ablation (CA) is an excellent treatment option for AF patients to maintain sinus rhythm and prevent recurrence [10]. However, it is crucial to select appropriate patients for CA with a careful, individualized assessment of the type of AF, symptom burden, left ventricular function, comorbidities and procedural risk profile. In general, patients with less comorbidities such as structural heart disease, diabetes, obesity, and hypertension, are suitable for undergoing the CA procedure [11]. 

Biomarkers play a significant role as risk predictors for some cardiovascular diseases [12]. In the case of AF, however, relatively limited evidence exists for usefulness of biomarkers to support patient managements. Troponin and brain natriuretic peptide have been expected to support prognosis prediction for AF patients, but further studies are warranted to demonstrate their clinical values [12]. 

MicroRNAs (miRNAs) represent a class of non-coding small RNAs with average lengths of 22-nucleotide. Most miRNAs act as negative regulators of target gene products via cleavage of messenger RNA (mRNA) or translational repression, some miRNAs can also upregulate target genes [13]. miRNAs play important roles in regulation of major physiological functions in the cardiovascular system such as atrial contractility, automaticity, ion channel performance, and aging processes [14,15,16]. In addition, miRNAs possess advantageous characteristics as biomarkers for clinical use [17], since they are remarkably stable in body fluids such as blood [18]. A number of miRNAs associated with AF have been reported both in heart tissue and circulating levels (reviewed in [14,19,20]). A key pathogenic factor for AF is left atrial remodeling. Moreover, advanced left atrial remodeling is associated with increased risk of thromboembolism, while less advanced one is associated with successful catheter ablation. Therefore, it would be meaningful to determine the particular types of miRNAs that are commonly associated with high/low thromboembolic risks and less/more performing catheter ablation, which may contribute to the decision-making in such important treatment strategies. Thus, the aim of the present study is to explore potential circulating miRNA biomarkers that are associated with CHA_2_DS_2_-VASc score and performing CA pursuing the quantitative measurement of the risk and the estimation of the mechanistic background.

## 2. Results

### 2.1. Patient Characteristics

Patient characteristics are presented in Table 1. Average ages of male and female patients were 67.4 and 74.0 years old, respectively. The number of patients with paroxysmal-, persistent- and permanent-type were 48 (57.8%), 13 (15.7%) and 22 (26.5%), respectively. The number of patients who underwent CA during the study period was 32, in which the numbers of patients with paroxysmal-, persistent- and permanent-type were 22 (68.8%), 9 (28.1%) and 1 (3.1%), respectively. Characteristics of patients who underwent catheter ablation is presented in Table 2.

### 2.2. Identification of Circulating miRNAs Potentially Associated with Prognosis Risks

**Figure 1:**
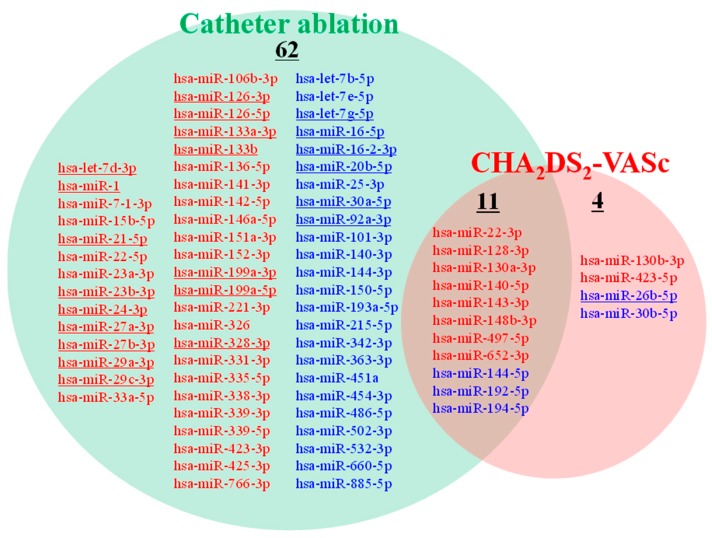
Circulating miRNAs whose plasma levels were correlated with CHA_2_DS_2_-VASc score and/or CA. miRNAs whose plasma levels showed positive (**red letters**) or negative (**blue letters**) correlation with CHA_2_DS_2_-VASc score (*q* < 0.1) are presented in the red circle. miRNAs whose plasma levels showed positive (blue letters) or negative (red letters) correlation with CA operation (*q* < 0.1) are presented in the green circle. Underlined miRNAs are reported to be cardiac-enriched miRNAs [21].

Ten and five miRNAs showed significant positive and negative correlations with CHA_2_DS_2_-VASc score, respectively (Figure 1). 

**Figure 2:**
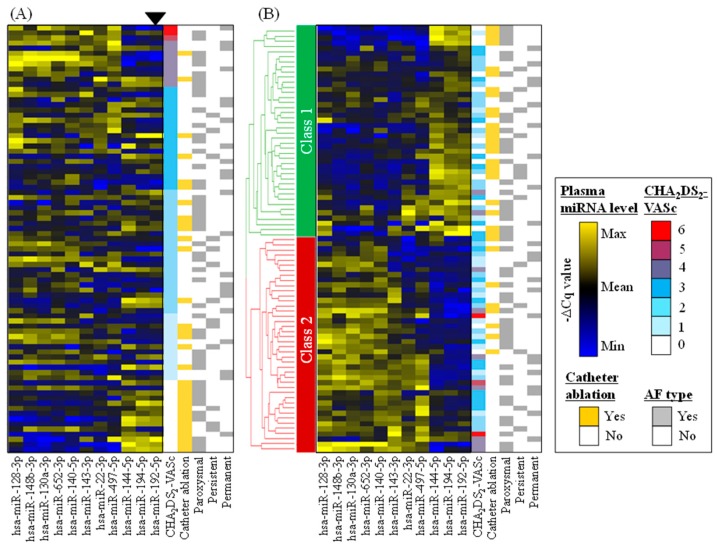
Plasma levels of miRNAs which were correlated with both CHA_2_DS_2_-VASc score and CA. Plasma levels (i.e., −∆C*q* values) of 11 miRNAs, whose plasma levels showed correlations with both CHA_2_DS_2_-VASc score and CA operation, are presented as heat maps. (**A**) The data was sorted with CHA_2_DS_2_-VASc score; (**B**) Plasma levels of the 11 miRNAs were subjected to hierarchical clustering. Based on the circulating miRNA profile, Class 1 and Class 2 patients are supposed to be associated with low and high stroke risks, respectively. The number of AF patients with paroxysmal-, persistent- and permanent-type were 48, 13 and 22, respectively, and 32 patients received CA operation during the study period. Note that plasma levels of the miRNA are high when their −∆C*q* is high. Yellow, black and blue colors represent high, middle and low expression levels of miRNAs, respectively. Red, blue and white colors represent high, middle and low CHA_2_DS_2_-VASc scores, respectively.

**Figure 3:**
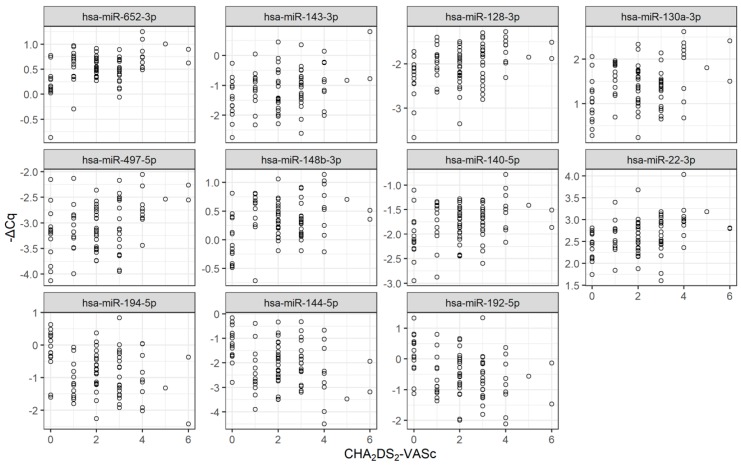
miRNAs associated with both CHA_2_DS_2_-VASc score and CA. Plasma levels (i.e., −∆C*q* values) of 11 miRNAs, whose plasma levels showed correlations with both CHA_2_DS_2_-VASc score and CA operation, are presented as dot plots. X and Y axis represent plasma level (−∆Cq value) and CHA_2_DS_2_-VASc score, respectively. Note that plasma levels of the miRNA are high when their −∆C*q* is high.

Forty-six and twenty-seven miRNAs showed significant increase and decrease in the patients who did not undergo CA compared to those who underwent CA. Eleven miRNAs were commonly identified as both CHA_2_DS_2_-VASc- and CA-associated miRNAs. Plasma levels (i.e., −∆C*q* value for each miRNA) of these 11 miRNAs are presented as heat map (Figure 2) and as dot plots (Figure 3). Eight miRNAs (i.e., hsa-miR-22-3p, hsa-miR-128-3p, hsa-miR-130a-3p, hsa-miR-140-5p, hsa-miR-143-3p, hsa-miR-148b-3p, hsa-miR-497-5p, hsa-miR-652-3p) tended to show higher plasma levels in patients with high CHA_2_DS_2_-VASc scores and lower plasma levels in patients who underwent CA (Figure 2A). Three miRNAs (i.e., hsa-miR-144-5p, hsa-miR-192-5p, hsa-miR-194-5p) tended to show lower plasma levels in patients with high CHA_2_DS_2_-VASc scores and higher plasma level in patients who underwent CA. Hierarchical clustering analysis on plasma miRNA levels (i.e., −∆C*q* of the miRNAs) classified the patients into two classes (Figure 2B), where patients with high risks (i.e., high CHA_2_DS_2_-VASc score and/or without CA operation) tended to be classified into Class 2, and patients with low risks (i.e., low CHA_2_DS_2_-VASc score and/or with CA operation) tended to be classified into Class 1. In addition to CHA_2_DS_2_-VASc score, we also investigated if there existed miRNAs whose plasma levels showed significant correlations to HAS-BLED score, which is clinically useful for predicting bleeding risk of AF patients [22], but it turned out that no such miRNAs existed in the present study. 

### 2.3. Pathway Enrichment Analysis

**Figure 4:**
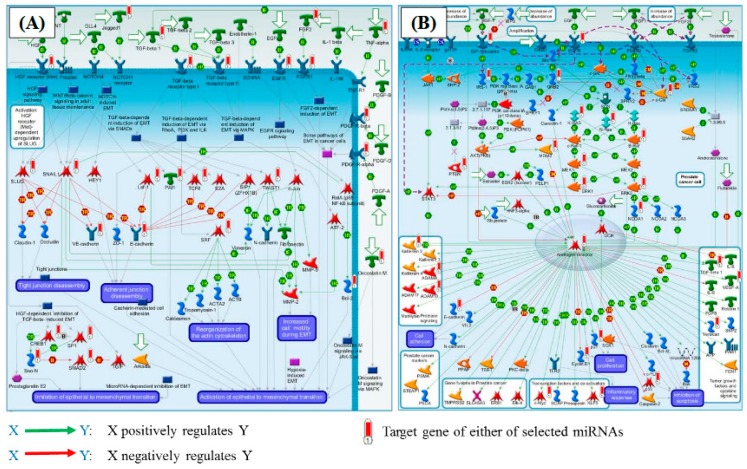
Representative pathways enriched with possible target genes of eight miRNAs whose plasma levels showed positive and negative correlations with CHA_2_DS_2_-VASc score and CA, respectively. (**A**) Pathway maps for “Development_Regulation of epithelial-to-mesenchymal transition (EMT)” and (**B**) “Pathway maps for “Androgen receptor activation and downstream signaling in Prostate cancer”, the 1st and 2nd most enriched pathways, respectively, querying possible target genes of the miRNA whose plasma levels showed positive correlations with CHA_2_DS_2_-VASc score (Table 3). Red bar represents possible target genes of miRNAs whose plasma levels were positively and negatively correlated with CHA_2_DS_2_-VASc score and CA, respectively. Green and red arrows mean positive and negative regulation of the target genes, respectively.

Among the 11 miRNAs selected as above, those whose plasma levels were positively and negatively correlated with CHA_2_DS_2_-VASc score were separately subjected to pathway enrichment analysis. The q-values for the enriched pathways were generally smaller in the results for miRNAs with positive correlation to CHA_2_DS_2_-VASc score compared with those with negative correlation to CHA_2_DS_2_-VASc score. The top 20 enriched pathways for positive and negative correlations to CHA_2_DS_2_-VASc score are presented in the Table 3 and Table 4, respectively, and both results showed similar enriched pathways such as epithelial-mesenchymal transition, sex hormone signaling, TGF-β signaling, and epigenetic alterations, etc. As representative pathways, pathway map for “Development_Regulation of epithelial-to-mesenchymal transition (EMT)” and “Androgen receptor activation and downstream signaling in Prostate cancer” provided in the Metabase knowledgebase are presented in Figure 4. These results should be interpreted with care since the source(s) of the circulating miRNAs have not been clarified at present. 

## 3. Discussion

### 3.1. Identification of 11 miRNAs Potentially Associated with Decision Making in AF Clinical Practice

The major objective of the study was to explore circulating miRNAs which can provide mechanistic and quantitative information for decision making in AF clinical practice, which identify the patients with high risk for ischemic stroke and the patients with good conditions for performing CA. We identified 15 miRNAs that showed significant correlations with CHA_2_DS_2_-VASc score (Figure 1), which is the most commonly used risk score of ischemic stroke in AF patients. Interestingly, eleven miRNAs of the fifteen were also included in the seventy-three miRNAs which showed correlation with CA operation during the follow-up period up to 6-month after enrollment (Figure 1). Of the eleven miRNAs, plasma levels of eight miRNAs (miR-22-3p, miR-128-3p, miR-130a-3p, miR-140-5p, miR-143-3p, miR-148b-3p, miR-497-5p, miR-652-3p) and three miRNAs (miR-144-5p, miR-192-5p and miR-194-5p) showed positive and negative correlations to CHA_2_DS_2_-VASc score, respectively. In contrast, the eight and the three miRNAs showed negative and positive correlations to CA operation, respectively. These 11 miRNA are not reported as cardiac-enriched miRNAs [21], and the source tissues of these miRNAs are not clear at this moment.

### 3.2. Possible Functional Implication Relevant to 11 miRNAs

To gain insights into functional implications for the dysregulation in circulating miRNA profile, pathway enrichment analysis was conducted for possible target genes of the 11 miRNAs. The results suggested that the possible target genes of the 11 miRNAs were functionally associated with TGF-β signaling, epithelial-mesenchymal transition, androgen signaling, etc. (Table 3 and Table 4). TGF-β signaling is a key pro-fibrotic element in various tissue and was reported to be capable of activating atrial fibroblasts to differentiate into myofibroblasts and promoting them to proliferate, migrate, and generate extracellular matrix protein [23,24], thus contributing to atrial fibrosis. It was reported that left atrial fibrosis is associated with an increased risk of thromboembolism in AF patients [25]. Endothelial-to-mesenchymal transition (EndMT) was reported to occur in atrium of AF patients [26], and TGF-b signaling also plays a key role in EndMT. EndMT has been reported to be strongly associated with thrombus formation in animal models of iliac vein compression syndrome [27], and deductively, EndMT would potentially affect stroke risk in AF patients. In addition, testosterone deficiency has been associated with the risk of AF in aging males [28], and testosterone therapy is related to thrombotic and cardiovascular events [29]. Collectively, results from the pathway enrichment analysis for possible target genes of dysregulated miRNAs were in good agreement with current knowledge on stroke risks in AF patients. 

### 3.3. Possible Target Genes Strongly Regulated by 11 miRNAs

**Figure 5:**
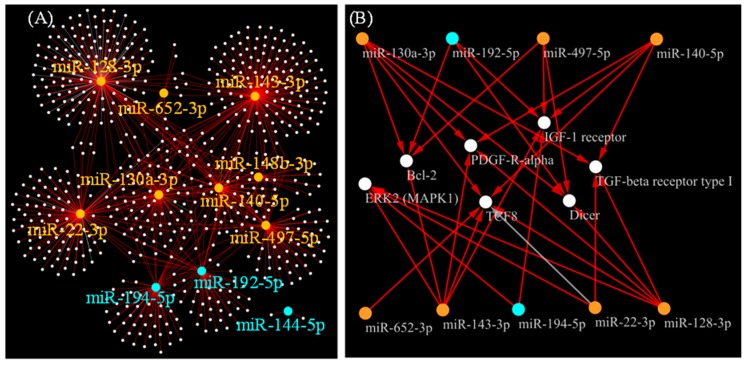
Network model representing miRNA-target gene relationship. (**A**) Network model representing miRNA-target gene interactions is presented, focusing on 11 miRNAs whose plasma levels showed correlations with both CHA_2_DS_2_-VASc score and CA operation, by referring to Metabase knowledgebase. White dots represent possible target genes. Orange and cyan dots represent miRNAs whose plasma levels showed positive and negative correlation with CHA_2_DS_2_-VASc score, respectively. Supporting literature information for the miRNA-target gene relationships is provided in Figure S1, and the network model file is provided as Figure S2; (**B**) Possible target genes that have ≥4 edges connected to either of the selected 11 miRNAs are presented. Orange and blue nodes indicate miRNAs whose plasma levels showed positive and negative correlations to CHA_2_DS_2_-VASc scores, respectively. Red and gray edges represent negative and unknown regulations of the miRNAs on their target genes, respectively.

To comprehend the relationships between the selected 11 miRNAs and target genes, we constructed a network model by referring to a commercial knowledgebase Metabase (Figure 5A). We further filtered genes connected to ≥4 miRNAs in the network model, resulting in identifying the seven genes: *TGFBR1*, *PDGFRA*, *TCF8* (*ZEB1*), *IGF1R*, *BCL2*, *MAPK1* and *DICER1* which were connected to either of nine miRNAs of the eleven (Figure 5B). 

Of the seven target genes, *TGFBR1*, *PDGFRA* and *ZEB1* are reported to be associated with fibrosis; e.g., TGF-β and PDGF are component of fibrotic pathways [30], ZEB1 was reported to mediate TGF-β signaling in vascular smooth muscle cell differentiation [31]. The up-regulations of seven circulating miRNAs (miR-22-3p, miR-128-3p, miR-130a-3p, miR-140-5p, miR-143-3p, miR-194-5p and miR-497-5p) in patients with high stroke risk (high CHA_2_DS_2_-VASc score) may suggest feedback regulations against pro-fibrotic condition in the patients. While we did not collect histological data, there exist possibility that dysregulation of these miRNAs may not stringently coincide with histological fibrosis, considering a previous report in mouse model that showed discordant observations for pro-fibrotic molecular signals and actual histological phenotypes [3], suggesting that certain time may be required for development of histologically detectable fibrosis after initiation of pro-fibrotic molecular signals. We speculate that molecular signals of pro-fibrotic condition would be more sensitive marker compared to histological fibrosis evaluated with imaging technologies, and useful for clinical risk management of AF patients, allowing early intervention against fibrosis. 

Besides the above three pro-fibrotic genes, *IGF1R*, *BCL2*, *MAPK1* and *DICER1* were also identified as possible target genes of the selected miRNAs. Contrary to the cases in three pro-fibrotic genes, *IGF1R*, *BCL2*, *MAPK1* and *DICER1* were connected to mixture of miRNAs which were either positively or negatively correlated to CHA_2_DS_2_-VASc score, suggesting complicated regulations of these genes by the miRNAs. This may reflect the stimulated functions of these genes associated with elevated stroke risks. It was reported that elderly AF patients show low level of serum IGF-1 level [32], and low level of circulating IGF-1 was associated with risk of ischemic stroke in AF patients, especially in diabetic and obese patients [33]. In addition, increased and decreased expression of atrial ERK1/2 and BCL2, respectively, were reported in AF patients [34,35]. ERK signaling is involved in cardiac hypertrophy, in which ERK1 and ERK2 have redundant functions [36], and it was reported that the ERK pathway acts to promote a compensated hypertrophic response and reduced fibrosis in the heart in a mice model overexpressing *ERK1* in cardiomyocyte [37]. BCL2 is an anti-apoptotic protein for heart fibroblast [38] and is reported to be potentially associated with myocardial fibrosis phenotype in patients with dilated cardiomyopathy (DCM) [39]. DICER1, an endonuclease required for processing of miRNAs were also identified as a candidate target gene connected to 4 miRNAs of the 11. This may reflect active regulations of miRNAs in the AF patients with high stroke risk, and it is also possible that dysregulation of DICER1 may be associated with cardiac pathology including fibrosis since considering that deletion of Dicer led to DCM and heart failure in mice [40]. Collectively, changes in plasma levels of the miRNAs would reflect elevated risk of stroke in AF patients, and could potentially serve as clinical biomarkers to support evaluating stroke risk of patients.

### 3.4. Clinical Implications: Classifying AF Patients Using 11 miRNAs Possibly Reflecting the Pro-Fibrotic State

Although CHA_2_DS_2_-VASc score is convenient and useful for stratifying patients with high stroke risk, it is a point-based score only taking account of selected risk factors of patients’ clinical characteristics and comorbidities and does not provide mechanistic and quantitative information to support evaluation of stroke risks. Meanwhile, careful patient selection is necessary for performing CA. According to the guidelines by Japanese Circulation Society, the most typical indication for performing CA is paroxysmal, symptomatic, drug-refractory AF without advanced left atrial enlargement or left ventricular dysfunction (Class I) [41,42]. This indication is based on the recognition that patients with the best outcome and the lowest procedural risk with CA are those with paroxysmal AF, structurally normal hearts and no comorbidities [43], while outcomes are less favorable for the cases of persistent AF [44]. However, considering such patient characteristics for patient selection may be similar to the “point-based scoring” because it does not provide mechanistic and quantitative information to support evaluation of the patient status.

The selected 11 miRNAs may potentially provide mechanistic and quantitative information for the significant pathophysiology in daily clinical practice with AF, possible pro-fibrotic state in left atrium, which would enhance the risk of ischemic stroke and reduce the preference for performing CA. For instance, plasma profiles of the 11 miRNAs can classify the patients into two clusters as shown in Figure 2B, where Class 1 and Class 2 patients would be associated with low and high risks, respectively. An intriguing hypothesis is that we may be able to judge high risk patients for ischemic stroke (i.e., Class 2 patients) or we may be able to select suitable patients for CA operation (i.e., Class 1 patients) based on plasma miRNA profile. Although we could find apparent relationships between the distribution of 11 miRNAs and thromboembolic risk score (CHA_2_DS_2_-VASc score) or clinical performance of CA, unfortunately, they were not the findings based on the direct relationship between the miRNAs and the prognosis (i.e., ischemic stroke or recurrence after CA). Therefore, more investigations will be necessary to further explore whether other miRNAs have similar predictive potentials, or to confirm that the 11 miRNAs we determined in the present study have actual predictive ability for the prognosis in a prospective manner. 

### 3.5. Limitations

A number of limitations should be considered. First, the number of patients may be too small to draw robust conclusions. Further evidence is needed to verify the present findings, and eventually prove the superiority of additional miRNA measurement to current clinical practice by conducting a prospective randomized controlled studies. 

Second, although a number of publications describe AF-associated miRNAs, considerable difference in their study designs hinders straightforward comparison among the studies [45]. While many of previous studies applied case-control comparisons (e.g., AF patients vs. healthy subjects), we only analyzed AF patient samples in the present study and identified miRNAs associated with stroke risk of AF patients, which therefore is not straightforwardly comparable with published information. Furthermore, the origin and function of miRNAs in tissue and plasma are usually unknown, and miRNA regulations in different tissues (e.g., cardiac tissue and blood) can frequently be contradicting results [20]. These factors need to be carefully considered to avoid misinterpretation of the findings. 

Third, from analytical point of views, optimization and standardization of data acquisition procedure will be essential, including conditions for blood collection, sample processing and storage, RNA extraction, miRNA measurement platform, etc. Regarding the miRNA data analysis process, no gold standard normalization methods for circulating miRNA panel data has been established. A global mean normalization is reported to be effective when sufficient number of target miRNAs are measured [46]. In addition, a guideline provided by Qiagen, the vendor of qRT-PCR used in the present investigation, recommended to use global mean normalization method [47]. Accordingly, we adopted this normalization method in the present study. 

Fourth, although pathway enrichment analysis has been frequently utilized to elucidate functional implications for dysregulated circulating miRNAs, this methodology cannot be free from inherent information bias and may provide inaccurate results [48]. In the present study, results from pathway enrichment analysis looked fairly consistent with AF pathophysiological mechanisms, however further supporting data will be necessary to verify the findings from the present study, not only with bioinformatics but with experimental approaches. At present, we need to note that our findings are still highly speculative, and further supporting evidence need to be accumulated from clinical researches to confirm associations of miRNA profiles and histological or MRI based verification on fibrotic conditions. In addition, as discussed above, tissue origin of the circulating miRNAs also needs to be better clarified with experimental approach to improve our understanding for dysregulation of circulating miRNA profiles. 

Lastly, to establish a practical clinical measurement system, sufficient level of dynamic range is desired with reasonable precision, accuracy, specificity and sensitivity, etc. However, altered levels of each miRNA among patients were very small in the present study, and therefore it would be technically challenging to measure the selected miRNAs individually. To address this difficulty, it would be practical to consider evaluating composite of the altered levels of the selected miRNAs, similar to the case in conducting “gene signature” analysis which provides better robust information compared with individual gene-level analysis [49]. 

## 4. Materials and Methods 

### 4.1. Ethics and Informed Consent

The current investigation is an exploratory sub-study conducted in an observational study enrolling non-valvular AF patients. The study was approved by the Institutional Review Board of the Cardiovascular Institute (1 May 2017) and was registered on the UMIN Clinical Trials Registry (UMIN 000028383, 26 Jul 2017). Study outline and results had been published previously [50,51]. The study was conducted in accordance with the ethical norms based on the Declaration of Helsinki (revised in 2013) and Ethical Guidance for Medical and Health Research Involving Human Subjects (Public Notice of the Ministry of Education, Culture, Sports, Science and Technology, and the Ministry of Health, Labour and Welfare, Japan, issued in 2017). Written informed consent was obtained from all participants. The study protocol was reviewed by the Institutional Review Board of the Cardiovascular Institute. 

### 4.2. Study Population

Patients with non-valvular AF receiving edoxaban (Daiichi Sankyo Co., Ltd., Tokyo, Japan), a direct oral anticoagulants, for at least 2 weeks were enrolled. The exclusion criteria were as follows: (1) receiving dual-anti-platelet therapy, (2) inadequate dosage of edoxaban at registration, (3) edoxaban hypersensitivity, (4) patients who are bleeding, (5) patients with acute bacterial endocarditis, (6) renal dysfunction (creatinine clearance  <30 mL/min), (7) liver dysfunction with clotting disorders, (8) patients with previous admission within 1 month before the registration for stroke, myocardial infarction, percutaneous coronary intervention, heart failure or bleeding, (9) patients who did not give written informed consent for this study, and (10) patients who are judged by the researchers as inadequate for this study. The patients were followed up for maximum of 6 months, and the number of patients who underwent catheter ablation during this period was recorded. Measurement of miRNA was done on samples from eighty-three AF patients (61 males and 22 females), all drawn in the morning to mitigate potential circadian rhythm of miRNA profiles. 

### 4.3. RNA Extraction and miRNA Measurement

Blood was drawn from patients, and plasma samples were prepared with EDTA-2K, and were stored at −30 to −10 C° until RNA extraction. miRNA was extracted from 200 μL plasma using the miRNeasy Mini Kit (Qiagen, Venlo, The Netherlands) according to the manufacturer’s instructions. Single-stranded cDNA was synthesized with Universal cDNA Synthesis Kit II, 8-64 rxns (Product No.: 203301, Qiagen, Venlo, Netherlands). A total of 179 circulating miRNAs were measured using Serum/Plasma Focus microRNA PCR Panel (Qiagen) and LightCycler^®^ 480 Instrument II (Roche Diagnostics, Basel, Switzerland). The Serum/Plasma Focus microRNA Panel contains 179 miRNA primer sets which had been selected based on the vendor’s vast number of in-house analysis as well as a number of publications [47]. The PCR panel primers contain seven candidate reference miRNAs that can also be used to assess RNA quality, hemolysis and contaminants, principle of which was described in a previous publication [52]. The Cq value, which represents the PCR cycle upon reaching the designated threshold amplification level, was determined for all target miRNAs using GenEx software (Qiagen) according to the manufacturer’s instructions. Target miRNAs whose amplification levels did not reach the designated threshold after the 40-cycle amplification were considered absent and were excluded from further analysis.

### 4.4. qRT-PCR Data Analysis

A global mean normalization method was adopted for normalizing the data [46]. In the present study, the C*q* values of miRNAs with C*q* < 37 were averaged and subtracted from all C*q* values for each sample. Data for two samples showed extremely deviated profile from other samples based on principal component analysis, and these two samples were excluded from further analysis. Differentially expressed miRNAs in each phenotype were calculated by fitting to the following linear model:

(miRNA expression) ~ (phenotype X) + (days_trough_sampling)

where phenotype represents each parameter or event, and days_trough_sampling represents the date of blood sampling for miRNA measurement, which can be an indicator of sample storage duration until RNA extraction. To select the miRNAs associated with CA and CHA_2_DS_2_-VASc scores, *q* < 0.1 were considered statistically significant. Hierarchical clustering analysis and heat map creation for the plasma miRNA levels was performed with TIBCO Spotfire^®^ (TIBCO Software Inc., Palo Alto, CA, USA). The clusters of the heat map were determined from a dendrogram generated by the UPGMA method using Euclidean distance. The top 50 abundant miRNAs in human heart reported by Liang et al. [21] are referred to as “cardiac-enriched miRNAs” in Figure 1. 

### 4.5. In Silico Functional Analysis

Metabase (Clarivate Analytics, Philadelphia, PA, USA) was used for the pathway enrichment analysis using the 11 miRNAs commonly identified as both CHA_2_DS_2_-VASc- and CA-correlated miRNAs. Metabase is a comprehensive manually curated data/ knowledgebase of mammalian biology and chemistry data, which have been utilized for systems biology analysis in various studies [53,54,55]. The Metabase version 19.2.69700, which includes 12,040 genes within 1502 canonical pathways. The enrichment *p*-value and the false discovery rate-adjusted *p*-value (*q*-value) for each of the pathways was generated using a hypergeometric test. The network model was visualized using Cytoscape software [56]. 

## 5. Conclusions

In conclusion, we identified circulating miRNAs whose plasma levels are potentially associated with high/low thromboembolic risks and less/more performing CA in AF patients. These miRNAs would provide mechanistic and quantitative information for pathophysiological conditions of AF patients, particularly associated with pro-fibrotic conditions, and would add values for decision making for administering anticoagulation therapy and performing CA. Further data is warranted to strengthen robustness of the present findings and to develop practical clinical biomarkers to support management of AF patients.

## Figures and Tables

**Table 1:** Characteristics of atrial fibrillation (AF) patients subjected to miRNA profiling.

Risk Scores	*n* = 83	%
Gender		
Male	61	73.5%
Female	22	26.5%
Age (mean ± SD)		
Male	67.4 ± 11.4	
Female	74.0 ± 7.2	
AF type		
Paroxysmal	48	57.8%
Persistent	13	15.7%
Permanent	22	26.5%
Number of Patients Underwent Catheter Ablation		
Paroxysmal	22	26.5%
Persistent	9	10.8%
Permanent	1	1.2%
CHADS_2_ Score		
0	25	30.1%
1	33	39.8%
2	15	18.1%
3	7	8.4%
4	3	3.6%
>5	0	0%
CHA_2_DS_2_-VASc Score		
0	14	16.9%
1	13	15.7%
2	24	28.9%
3	20	24.1%
4	9	10.8%
5	1	1.2%
6	2	2.4%
>7	0	0%
HAS-BLED Score		
0	17	20.5%
1	25	30.1%
2	30	36.1%
3	11	13.3%
>4	0	0%

**Table 2:** Characteristics of patients who underwent catheter ablation.

	Total (*n* = 33)	Paroxysmal AF (*n* = 22)	Persistent AF (*n* = 10)	Permanent AF (*n* = 1)
Age, years	61.3 ± 10.7	62.3 ± 11.1	59.7 ± 10.3	56
Male, *n* (%)	28 (85)	19 (86)	8 (80)	1
CHA_2_DS_2_-VASc, points	1 (0–2)	1 (0–2)	1 (0–2)	0
EHRA Score				
1	12 (36)	6 (27)	5 (50)	1
2	16 (49)	12 (55)	4 (40)	0
3	2 (6)	1 (5)	1 (10)	0
4	3 (9)	3 (14)	0 (0)	0
Mitral regurgitation, *n* (%)	0 (0)	0 (0)	0 (0)	0
Mitral stenosis, *n* (%)	0 (0)	0 (0)	0 (0)	0
Aortic regurgitation, *n* (%)	0 (0)	0 (0)	0 (0)	0
Aortic stenosis, *n* (%)	1 (3)	1 (5)	0 (0)	0
LVEF, %	63.3 ± 8.8	63.9 ± 9.9	61.3 ± 5.8	72
LAVI, mL/m^2^	39.3 ± 17.7	38.8 ± 20.4	37.7 ± 10.9	61
Recurrence within 1 year	1 (3)	1 (5)	0 (0)	0

AF, atrial fibrillation; LVEF, left ventricular ejection fraction; LAVI, left atrial volume index.

**Table 3:** Pathway enrichment analysis using possible target genes of the miRNA whose plasma levels showed positive correlations with CHA_2_DS_2_-VASc score.

Pathways	Number of Mapped Genes	Number of Genes in the Pathway	*q*-Value
Development_regulation of epithelial-to-mesenchymal transition (EMT)	26	64	7.8 × 10^−20^
Androgen receptor activation and downstream signaling in prostate cancer	31	110	8.0 × 10^−19^
Development_TGF-β receptor signaling	23	52	8.0 × 10^−19^
Dual role of TGF-β 1 in HCC	17	24	1.7 × 10^−18^
Development_YAP/TAZ-mediated co-regulation of transcription	23	56	3.8 × 10^−18^
TGF-β signaling via SMADs in breast cancer	21	47	1.8 × 10^−17^
Main genetic and epigenetic alterations in lung cancer	21	48	2.7 × 10^−17^
Cell cycle_Regulation of G1/S transition (part 1)	19	38	5.4 × 10^−17^
TGF-β 1-mediated induction of EMT in normal and asthmatic airway epithelium	20	44	6.1 × 10^−17^
Mechanisms of resistance to EGFR inhibitors in lung cancer	20	45	9.6 × 10^−17^
Ligand-independent activation of androgen receptor in prostate cancer	23	67	2.1 × 10^−16^
Inhibition of TGF-β signaling in lung cancer	17	31	3.5 × 10^−16^
Activation of TGF-β signaling in pancreatic cancer	16	27	5.9 × 10^−16^
K-RAS signaling in pancreatic cancer	19	44	1.0 × 10^−15^
Main pathways of Schwann cells transformation in neurofibromatosis type 1	24	80	1.0 × 10^−15^
TGF-β signaling via kinase cascades in breast cancer	21	58	1.3 × 10^−15^
Role of microRNAs in cell migration, survival and angiogenesis in colorectal cancer	22	67	2.4 × 10^−15^
Stromal-epithelial interaction in prostate cancer	18	42	7.0 × 10^−14^
EGFR family signaling in pancreatic cancer	22	75	3.2 × 10^−14^
Role of microRNAs in cell proliferation in colorectal cancer	21	69	6.2 × 10^−14^

Pathway enrichment analysis was conducted with possible target genes regulated by eight miRNAs whose plasma levels were positively correlated with CHA_2_DS_2_-VASc score and negatively correlated with CA operation. The most significant 20 pathways in terms of *q*-values were presented.

**Table 4:** Pathway enrichment analysis using possible target genes of the miRNA whose plasma levels showed negative correlations with CHA_2_DS_2_-VASc score.

Pathways	Number of Mapped Genes	Number of Genes in the Pathway	*q*-Value
Androgen receptor activation and downstream signaling in prostate cancer	11	110	2.1× 10^−7^
TGF-β signaling via kinase cascades in breast cancer	8	58	2.9 × 10^−6^
Mitogenic action of ESR1 (membrane) in breast cancer	7	47	9.7 × 10^−6^
Main genetic and epigenetic alterations in lung cancer	7	48	9.7 × 10^−6^
IGF-1 signaling in multiple myeloma	7	50	1.0 × 10^−5^
SHH signaling in melanoma	6	33	1.3 × 10^−5^
IGF family, invasion and metastasis in colorectal cancer	6	33	1.3 × 10^−5^
Aberrant B-Raf signaling in melanoma progression	7	55	1.3 × 10^−5^
Signal transduction_AKT signaling	6	43	5.2 × 10^−5^
Mechanisms of drug resistance in SCLC	7	70	5.2 × 10^−5^
Development_Ligand-independent activation of ESR1 and ESR2	6	44	5.2 × 10^−5^
Stem cells_stimulation of differentiation of mouse embryonic fibroblasts into adipocytes by extracellular factors	7	71	5.2 × 10^−5^
Immune response_IL-2 signaling via ERK, PI3K, and PLC-γ	7	73	5.8 × 10^−5^
Neuroendocrine transdifferentiation in prostate cancer	6	48	7.5 × 10^−5^
The role of PTEN and PI3K signaling in melanoma	6	50	9.0 × 10^−5^
Anti-apoptotic action of ErbB2 in breast cancer	6	51	9.5 × 10^−5^
Stem cells role of growth factors in the maintenance of embryonic stem cell pluripotency	6	53	1.1 × 10^−4^
DNA damage_Brca1 as a transcription regulator	5	30	1.2 × 10^−4^
Cell adhesion_ECM remodeling	6	55	1.3 × 10^−4^
Putative role of estrogen and androgen receptor signaling in progression of lung cancer	6	58	1.6 × 10^−4^

Pathway enrichment analysis was conducted with possible target genes regulated by three miRNAs whose plasma levels were negatively correlated with CHA_2_DS_2_-VASc score and positively correlated with CA operation. The most significant 20 pathways in terms of *q*-values were presented.

## Supplementary Materials

Supplementary materials can be found at https://www.mdpi.com/1422-0067/21/7/2444/s1.

## Abbreviations

AF	Atrial fibrillation
CA	Catheter ablation
EHRA	European Heart Rhythm Association
EndMT	Epithelial-to-mesenchymal transition
LAVI	Left atrial volume index
LVEF	Left ventricular ejection fraction
mRNA	Messenger RNA
miRNA	MicroRNA
PDGFRA	PDGF-R-alpha
TGFBR1	TGF-β receptor type I

## References

[1] Schotten U., Verheule S., Kirchhof P., Goette A. (2011). Pathophysiological Mechanisms of Atrial Fibrillation: A Translational Appraisal. Physiol. Rev..

[2] De Jong A.M., Maass A.H., Oberdorf-Maass S.U., Van Veldhuisen D.J., Van Gilst W.H., Van Gelder I.C. (2010). Mechanisms of atrial structural changes caused by stretch occurring before and during early atrial fibrillation. Cardiovasc. Res..

[3] De Jong A.M., Van Gelder I.C., Vreeswijk-Baudoin I., Cannon M.V., Van Gilst W.H., Maass A.H. (2013). Atrial Remodeling Is Directly Related to End-Diastolic Left Ventricular Pressure in a Mouse Model of Ventricular Pressure Overload. PLoS ONE.

[4] Staerk L., Sherer J.A., Ko D., Benjamin E.J., Helm R.H. (2017). Atrial Fibrillation: Epidemiology, Pathophysiology, and Clinical Outcomes. Circ. Res..

[5] Rawal A., Ardeshna D., Minhas S., Cave B., Ibeguogu U., Khouzam R. (2019). Current status of oral anticoagulant reversal strategies: A review. Ann. Transl. Med..

[6] Lip G.Y., Nieuwlaat R., Pisters R., Lane D.A., Crijns H.J. (2010). Refining clinical risk stratification for predicting stroke and thromboembolism in atrial fibrillation using a novel risk factor-based approach: The euro heart survey on atrial fibrillation. Chest.

[7] Neefs J., Klamer T.A., Krul S.P.J., De Groot J.R. (2019). Should Every Patient with Atrial Fibrillation and a CHA_2_DS_2_-VASc Score of 1 Be Anticoagulated? A Systematic Review of 37,030 Patients. Cardiol. Rev..

[8] Kirchhof P., Benussi S., Kotecha D., Ahlsson A., Atar D., Casadei B., Castellà M., Diener H.-C., Heidbuchel H., Hendriks J. (2016). 2016 ESC Guidelines for the management of atrial fibrillation developed in collaboration with EACTS. Eur. Heart J..

[9] Apostolakis S., Guo Y., Lane D.A., Buller H., Lip G.Y.H. (2013). Renal function and outcomes in anticoagulated patients with non-valvular atrial fibrillation: The AMADEUS trial. Eur. Heart J..

[10] Asad Z.U.A., Yousif A., Khan M.S., Al-Khatib S.M., Stavrakis S. (2019). Catheter Ablation Versus Medical Therapy for Atrial Fibrillation. Circ. Arrhythmia Electrophysiol..

[11] Walsh K., Marchlinski F.E. (2018). Catheter ablation for atrial fibrillation: Current patient selection and outcomes. Expert Rev. Cardiovasc. Ther..

[12] Ardhianto P., Yuniadi Y. (2019). Biomarkers of atrial fibrillation: Which one is a true marker?. Cardiol. Res. Pract..

[13] Vasudevan S., Tong Y., Steitz J.A. (2007). Switching from repression to activation: MicroRNAs can up-regulate translation. Science.

[14] Santulli G., Iaccarino G., DeLuca N., Trimarco B., Condorelli G. (2014). Atrial fibrillation and microRNAs. Front. Physiol..

[15] Wang Z., Lu Y., Yang B. (2010). MicroRNAs and atrial fibrillation: New fundamentals. Cardiovasc. Res..

[16] De Lucia C., Komici K., Borghetti G., Femminella G.D., Bencivenga L., Cannavo A., Corbi G., Ferrara N., Houser S.R., Koch W.J. (2017). microRNA in Cardiovascular Aging and Age-Related Cardiovascular Diseases. Front. Med..

[17] Lopez J.P., Diallo A., Cruceanu C., Fiori L.M., LaBoissière S., Guillet I., Fontaine J., Ragoussis J., Benes V., Turecki G. (2015). Biomarker discovery: Quantification of microRNAs and other small non-coding RNAs using next generation sequencing. BMC Med. Genom..

[18] Brase J.C., Wuttig D., Kuner R., Sueltmann H. (2010). Serum microRNAs as non-invasive biomarkers for cancer. Mol. Cancer.

[19] Komal S., Yin J.-J., Wang S.-H., Huang C.-Z., Tao H.-L., Dong J.-Z., Han S.-N., Zhang L. (2019). MicroRNAs: Emerging biomarkers for atrial fibrillation. J. Cardiol..

[20] Van den Berg N.W.E., Kawasaki M., Berger W.R., Neefs J., Meulendijks E., Tijsen A.J., de Groot J.R. (2017). MicroRNAs in Atrial Fibrillation: From Expression Signatures to Functional Implications. Cardiovasc. Drugs Ther..

[21] Liang Y., Ridzon D., Wong L., Chen C. (2007). Characterization of microRNA expression profiles in normal human tissues. BMC Genom..

[22] Lane D.A., Lip G.Y. (2012). Use of the CHA(2)DS(2)-VASc and HAS-BLED scores to aid decision making for thromboprophylaxis in nonvalvular atrial fibrillation. Circulation.

[23] Burstein B., Nattel S. (2008). Atrial Fibrosis: Mechanisms and Clinical Relevance in Atrial Fibrillation. J. Am. Coll. Cardiol..

[24] Chang S.H., Yeh Y.H., Lee J.L., Hsu Y.J., Kuo C.T., Chen W.J. (2017). Transforming growth factor-beta-mediated CD44/STAT3 signaling contributes to the development of atrial fibrosis and fibrillation. Basic Res. Cardiol..

[25] Daccarett M., Badger T.J., Akoum N., Burgon N.S., Mahnkopf C., Vergara G., Kholmovski E., McGann C.J., Parker D., Brachmann J. (2011). Association of Left Atrial Fibrosis Detected by Delayed-Enhancement Magnetic Resonance Imaging and the Risk of Stroke in Patients with Atrial Fibrillation. J. Am. Coll. Cardiol..

[26] Kato T., Sekiguchi A., Sagara K., Tanabe H., Takamura M., Kaneko S., Aizawa T., Fu L.-T., Yamashita T. (2017). Endothelial–mesenchymal transition in human atrial fibrillation. J. Cardiol..

[27] Hong L., Du X., You T., Sun L., Li W., Xiao L., Lu H., Wang W., Li X. (2019). Reciprocal enhancement of thrombosis by endothelial-to-mesenchymal transition induced by iliac vein compression. Life Sci..

[28] Magnani J.W., Moser C.B., Murabito J.M., Sullivan L., Wang N., Ellinor P.T., Vasan R.S., Benjamin E.J., Coviello A.D. (2014). Association of sex hormones, aging, and atrial fibrillation in men: The Framingham Heart Study. Circ. Arrhythmia Electrophysiol..

[29] Glueck C.J., Wang P. (2014). Testosterone therapy, thrombosis, thrombophilia, cardiovascular events. Metabolism.

[30] Dzeshka M.S., Lip G.Y., Snezhitskiy V., Shantsila E. (2015). Cardiac Fibrosis in Patients with Atrial Fibrillation: Mechanisms and Clinical Implications. J. Am. Coll. Cardiol..

[31] Nishimura G., Manabe I., Tsushima K., Fujiu K., Oishi Y., Imai Y., Maemura K., Miyagishi M., Higashi Y., Kondoh H. (2006). DeltaEF1 mediates TGF-beta signaling in vascular smooth muscle cell differentiation. Dev. Cell.

[32] Duron E., Vidal J.-S., Funalot B., Brunel N., Viollet C., Seux M.-L., Treluyer J.-M., Epelbaum J., Le Bouc Y., Hanon O. (2013). Insulin-Like Growth Factor I, Insulin-like Growth factor Binding Protein 3, and Atrial Fibrillation in the Elderly. J. Gerontol. Ser. A Biol. Sci. Med. Sci..

[33] Saber H., Himali J.J., Beiser A., Shoamanesh A., Pikula A., Roubenoff R., Romero J.R., Kase C.S., Vasan R.S., Seshadri S. (2017). Serum Insulin-Like Growth Factor 1 and the Risk of Ischemic Stroke: The Framingham Study. Stroke.

[34] Goette A., Staack T., Röcken C., Arndt M., Geller J.C., Huth C., Ansorge S., Klein H.U., Lendeckel U. (2000). Increased expression of extracellular signal-regulated kinase and angiotensin-converting enzyme in human atria during atrial fibrillation. J. Am. Coll. Cardiol..

[35] Diao S.-L., Xu H.-P., Zhang B., Ma B.-X., Liu X.-L. (2016). Associations of MMP-2, BAX, and Bcl-2 mRNA and Protein Expressions with Development of Atrial Fibrillation. Med. Sci. Monit..

[36] Gallo S., Vitacolonna A., Bonzano A., Comoglio P.M., Crepaldi T. (2019). ERK: A Key Player in the Pathophysiology of Cardiac Hypertrophy. Int. J. Mol. Sci..

[37] Mutlak M., Schlesinger-Laufer M., Haas T., Shofti R., Ballan N., Lewis Y.E., Zuler M., Zohar Y., Caspi L.H., Kehat I. (2018). Extracellular signal-regulated kinase (ERK) activation preserves cardiac function in pressure overload induced hypertrophy. Int. J. Cardiol..

[38] Mayorga M., Bahi N., Ballester M., Comella J.X., Sanchís D., Junutula J.R., Schonteich E., Wilson G.M., Peden A.A., Scheller R.H. (2004). Bcl-2 Is a Key Factor for Cardiac Fibroblast Resistance to Programmed Cell Death. J. Biol. Chem..

[39] Wang Y., Li M., Xu L., Liu J., Wang D., Li Q., Wang L., Li P., Chen S., Liu T. (2016). Expression of Bcl-2 and microRNAs in cardiac tissues of patients with dilated cardiomyopathy. Mol. Med. Rep..

[40] Chen J.-F., Murchison E.P., Tang R., Callis T.E., Tatsuguchi M., Deng Z., Rojas M., Hammond S.M., Schneider M.D., Selzman C.H. (2008). Targeted deletion of Dicer in the heart leads to dilated cardiomyopathy and heart failure. Proc. Natl. Acad. Sci. USA.

[41] Japanese Circulation Society Joint Working Group (2018). JCS/JHRS Guideline on Non-Pharmacotherapy of Cardiac Arrhythmias. http://www.j-circ.or.jp/guideline/pdf/JCS2018_kurita_nogami.pdf.

[42] January C.T., Wann L.S., Alpert J.S., Calkins H., Cigarroa J.E., Cleveland J.C., Conti J.B., Ellinor P.T., Ezekowitz M.D., Field M.E. (2014). 2014 AHA/ACC/HRS guideline for the management of patients with atrial fibrillation: A report of the American College of Cardiology/American Heart Association Task Force on practice guidelines and the Heart Rhythm Society. Circulation.

[43] Lee G., Sparks P.B., Morton J.B., Kistler P.M., Vohra J.K., Medi C., Rosso R., Teh A., Halloran K., Kalman J.M. (2010). Low Risk of Major Complications Associated with Pulmonary Vein Antral Isolation for Atrial Fibrillation: Results of 500 Consecutive Ablation Procedures in Patients with Low Prevalence of Structural Heart Disease From a Single Center. J. Cardiovasc. Electrophysiol..

[44] Rostock T., Salukhe T.V., Steven D., Drewitz I., Hoffmann B.A., Bock K., Servatius H., Müllerleile K., Sultan A., Gosau N. (2011). Long-term single- and multiple-procedure outcome and predictors of success after catheter ablation for persistent atrial fibrillation. Heart Rhythm.

[45] Shen N.N., Zhang Z.L., Li Z., Zhang C., Li H., Wang J.L., Wang J., Gu Z.C. (2019). Identification of microRNA biomarkers in atrial fibrillation: A protocol for systematic review and bioinformatics analysis. Medicine.

[46] Mestdagh P., Van Vlierberghe P., De Weer A., Muth D., Westermann F., Speleman F., Vandesompele J. (2009). A novel and universal method for microRNA RT-qPCR data normalization. Genome Biol..

[47] Qiagen, Guidelines for Profiling Biofluid miRNAs. https://www.qiagen.com/us/-/media/project/qiagen/qiagen-home/documents/content-worlds/liquid-biopsy/guidelines-for-profiling-biofluid-mirnas.pdf.

[48] Godard P., van Eyll J. (2015). Pathway analysis from lists of microRNAs: Common pitfalls and alternative strategy. Nucleic Acids Res..

[49] Kiyosawa N., Manabe S., Yamoto T., Sanbuissho A. (2010). Practical Application of Toxicogenomics for Profiling Toxicant-Induced Biological Perturbations. Int. J. Mol. Sci..

[50] Suzuki S., Morishima Y., Takita A., Yagi N., Otsuka T., Arita T., Yamashita T. (2019). Responses of prothrombin time and activated partial thromboplastin time to edoxaban in Japanese patients with non-valvular atrial fibrillation: Characteristics of representative reagents in Japan (CVI ARO 7). Heart Vessels.

[51] Suzuki S., Morishima Y., Takita A., Otsuka T., Yagi N., Arita T., Yamashita T. (2020). Association between plasma concentration of edoxaban determined by direct and indirect methods in Japanese patients with non-valvular atrial fibrillation (CVI ARO 7). Heart Vessels.

[52] Blondal T., Nielsen S.J., Baker A., Andreasen D., Mouritzen P., Teilum M.W., Dahlsveen I.K. (2013). Assessing sample and miRNA profile quality in serum and plasma or other biofluids. Methods.

[53] Costa Sa A.C.C., Madsen H., Brown J.R. (2019). Shared Molecular Signatures Across Neurodegenerative Diseases and Herpes Virus Infections Highlights Potential Mechanisms for Maladaptive Innate Immune Responses. Sci. Rep..

[54] Godard P., Page M. (2016). PCAN: Phenotype consensus analysis to support disease-gene association. BMC Bioinform..

[55] Wang Z., Arat S., Magid-Slav M., Brown J.R. (2018). Meta-analysis of human gene expression in response to Mycobacterium tuberculosis infection reveals potential therapeutic targets. BMC Syst. Biol..

[56] Shannon P., Markiel A., Ozier O., Baliga N.S., Wang J.T., Ramage D., Amin N., Schwikowski B., Ideker T. (2003). Cytoscape: A software environment for integrated models of biomolecular interaction networks. Genome Res..

